# Polo-like kinase 4: A molecular linchpin in cancer and its management

**DOI:** 10.1016/j.isci.2025.114186

**Published:** 2025-11-22

**Authors:** Durdana Muntaqua, Gagan Chhabra, Karla B. Anaya Aldrete, Nihal Ahmad

**Affiliations:** 1 Department of Dermatology, University of Wisconsin, Madison, WI 53705, USA; 2 William S. Middleton Memorial Veterans Hospital, Madison, WI 53705, USA

**Keywords:** natural sciences, molecular biology, cell biology

## Abstract

Genomic instability and cell cycle dysregulation are considered hallmarks of cancer. Polo-like kinase 4 (PLK4), a member of the PLK family, is essential for faithful centriole duplication, which, when dysregulated, contributes to genomic instability, cell cycle disruption, and cancer development. PLK4 overexpression has been correlated with progression, metastasis, and poor patient survival in multiple cancers. However, the in-depth understanding of signaling pathways and the regulation of PLK4 in cancers continues to evolve. Similarly, the strategy of PLK4 inhibition for cancer management is currently being actively investigated. This review discusses the existing knowledge on the role and function of PLK4 and its relationship with genomic instability and cancer. Additionally, we have summarized studies showing the association of PLK4 with multiple cancers and how its modulation affects cancer progression. Further, we have discussed PLK4 inhibitors and molecular pathways that could be associated with PLK4 and can open new avenues in cancer management.

## Graphical abstract

**Figure:**
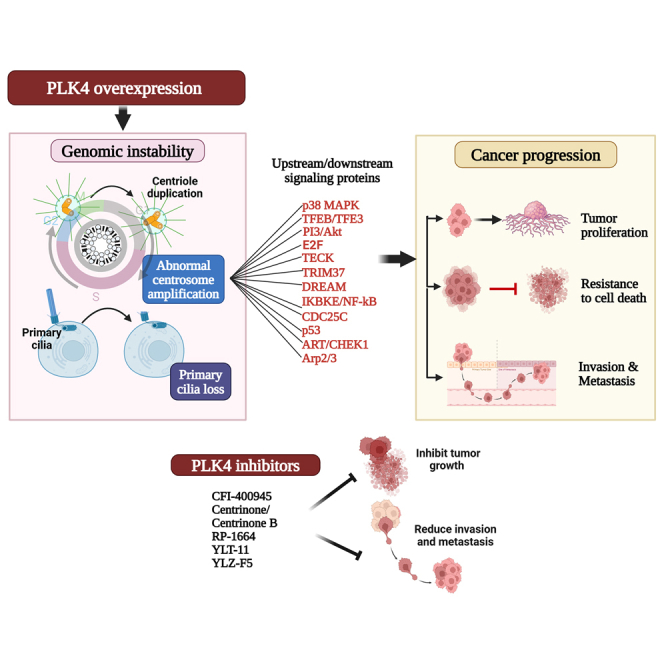

## Introduction

Genomic stability is crucial to maintain cellular homeostasis, prevent DNA replication errors during the cell cycle, and protect the cells against internal stress, such as from reactive oxygen species generated by cell metabolism and external carcinogens, for example, the ultraviolet (UV) radiation.^1^ Genomic instability allows damaged cells to have shorter cell cycles and evade various intracellular and immune regulatory mechanisms, enabling neoplastic transformation.^1^ The cell cycle is regulated by a complex system of kinases, among which polo-like kinases (PLKs) are critical for centriole duplication, spindle function, mitosis, and cytokinesis.^2^ The aberrant function of these kinases prompts genomic instability and uncontrolled cell division, leading to cancer.^3^ The PLK family encompasses five members (PLK 1–5), which are crucial in regulating multiple functions of the cell cycle, response to DNA damage, and apoptosis.^4^ In the last two decades, PLK4 has earned attention due to its potential involvement in multiple cancers.^5^^,^^6^^,^^7^^,^^8^^,^^9^^,^^10^^,^^11^^,^^12^ PLK4 is pivotal for centriole duplication and is considered the master regulator of centrosome amplification.^13^ Studies have shown an association between PLK4 overexpression and cancer progression, metastasis, resistance to chemotherapy, and overall poor patient survival.^5^^,^^6^^,^^7^^,^^8^^,^^9^^,^^10^^,^^11^^,^^12^ Consequently, PLK4 has emerged as a viable therapeutic target for cancer management, with ongoing clinical trials of PLK4 inhibitors for the treatment of cancer.^14^^,^^15^^,^^16^^,^^17^^,^^18^^,^^19^^,^^20^^,^^21^^,^^22^ However, limited information is available on the molecular mechanisms and crosstalk of PLK4 with other oncogenic pathways. Here, we have briefly discussed the structure and function of PLK4, its association in various cancers and its mechanisms. We have also discussed the potential of PLK4 as a therapeutic target against cancer and the preclinical and clinical studies on PLK4 inhibitors in cancer. Finally, we have provided a perspective on role of PLK4 signaling in cancer and the potential avenues that may be the subject of analysis for future studies.

## PLK4 structure, its regulation, and role in centriole duplication

### Structure

**Figure 1:**
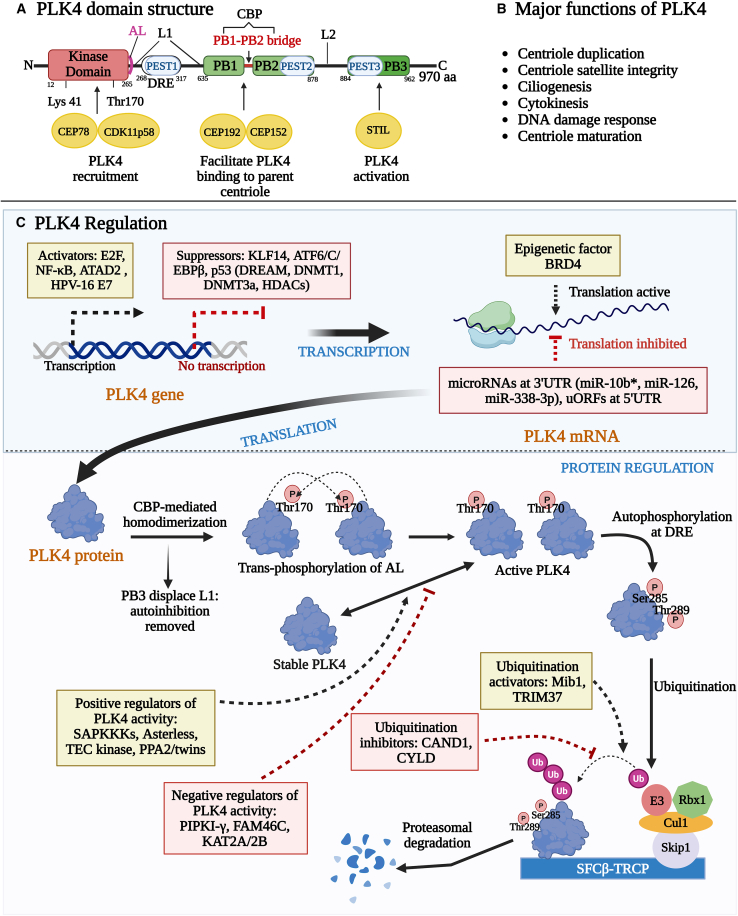
PLK4 structure, function, and regulation (A) Schematic representation of PLK4 domains. PLK4 protein has a kinase domain (KD), a cryptic polo-box (CPB) composed of PB1 and PB2, and a third polo-box PB3 domain. PB1 and PB2 are joined together by a bridge (red line). There are two linker regions L1 and L2. KD also has an activation loop (AL; purple) for its activation. There are three PEST regions and a DNA response element (DRE) that regulates kinase activity. These domains interact with proteins such as CEP78, CDK11p58, CEP192, CEP152, and STIL that recruit and activate PLK4 for centriole duplication. CEP192 and CEP152 facilitate the binding of PLK4 to parent centriole. (B) Major functions of PLK4 in centriole biogenesis, ciliogenesis, and DNA damage response. (C) Regulation of PLK4 at transcription, translation, and protein levels. PLK4 transcription is activated by E2F, NF-κB, ATAD2, and HPV-16 E7 whereas it is suppressed by KLF14, ATF6/C/EBPβ, and p-53-mediated activity of DREAM complex, DNA methyltransferases (DNMT1 and DNMT3a) and histone deacetylases (HDACs). PLK4 translation is known to be positively regulated by epigenetic factor BRD4 and negatively regulated by microRNAs (miR-10b∗, miR-126, and miR-338-3p) at 3′-untranslated region (UTR) and upstream open reading frames (uORFs) located in the 5′-UTR in PLK4 mRNA. Once the protein is synthesized it is regulated by trans/auto-phosphorylation at its various sites that either activates PLK4 or leads to proteasomal degradation of active PLK4. PLK4 ubiquitination is promoted by Mib1 and TRIM37 while it is inhibited by CAND1 and CYLD. Other regulators that activate or stabilize PLK4 include SAPKKKs, Asterless, TEC kinase and PPA2/twins. Negative regulators of PLK4 activity include PIPKI-γ, FAM46C and KAT2A/2B. Created in BioRender. Muntaqua, D. (2025) https://BioRender.com/grifshd.

The structure and functions of PLK4 have been previously described in multiple publications,^23^^,^^24^^,^^25^ and are summarized in Figures 1A and 1B. The human PLK4 protein (∼109 kDa) consists of 970 amino acids and features unique structural domains compared to other PLKs. It has a cryptic polo-box homodimer (CPB; combination of PB1 and PB2) beside a conserved polo-box (PB3) at the carboxy-terminal, which stabilized PLK4.^23^ In the kinase domain (KD), there are multiple amino acid residues that regulate the activity of PLK4.^26^ For example, Glu96 site contributes to the structural integrity and proper alignment of the catalytic domain.^26^ Thr170 is the key auto-phosphorylation site residing in the activation loop (AL) of PLK4, which is required for full kinase activation.^26^ Lys41 is the critical ATP-binding residue within the kinase domain that is necessary for transferring phosphate groups during phosphorylation of PLK4 substrates.^26^ In addition, the highly conserved sequences including HRD (His-Arg-Asp) in catalytic loop and DFG (Asp-Phe-Gly) in AL are essential for phosphotransferase activity and conformational switching between active/inactive states of PLK4, respectively.^27^^,^^28^ Besides, there are three composites (PEST) of proline (P), aspartate (D), glutamate (E), serine (S), and threonine (T) residues, one within the amino-terminus and two within the carboxy terminus of the kinase. These PEST sequences regulate stability of the kinase and turnover of PLK4 protein.^26^ Additional structural features include two linker regions (L1 and L2) and a PB1-PB2 bridge, which are involved in auto-regulation of PLK4 protein.^29^

### PLK4 regulation

PLK4 is regulated at transcriptional, translational and post-translational levels by multiple regulators,^30^^,^^31^^,^^32^^,^^33^^,^^34^^,^^35^^,^^36^^,^^37^^,^^38^^,^^39^^,^^40^ which work together to control centriole duplication (Figure 1C). Abnormal functions of these regulators may contribute to centrosome amplification, a phenomenon often observed in cancer. *PLK4* transcription is closely regulated by various transcription factors.^30^^,^^31^^,^^32^ Some of these transcriptional activators, such as E2F, NF-κB, ATAD2 etc. regulate *PLK4* expression and are associated with centrosome amplification in cancer (Figure 1C).^30^^,^^31^^,^^32^ E2F directly binds the P*LK4* promoter between exons 1 and 2 and enhances *PLK4* transcription.^32^ Similarly, the transcription factor NF-κB, positively influences P*LK4* gene expression.^31^ NF-κB subunits are shown to directly bind to *PLK4* promoter and increase P*LK4* mRNA levels. It was observed that knockdown of *NF-κB* in human dermal fibroblasts, osteosarcoma, and HeLa cells resulted in the loss of PLK4 expression.^31^ Furthermore, PLK4 was found to induce NF-κB transactivation by phosphorylating IKBKE, which in turn phosphorylates the inhibitor IκB to activate NF-κB signaling.^41^ ATAD2, an ATPase domain-containing protein, is also known to upregulate *PLK4* transcription, which was demonstrated in glioblastoma (GB) cells. It was suggested that the ATAD2-dependent transcriptional regulation of *PLK4* was essential for tumor growth and treatment resistance in GBM cells.^42^ These effects were potentially dependent on the induction of centrosome amplification and genomic instability, which are related to cancer pathogenesis.

Conversely, P*LK4* can be transcriptionally repressed by KLF14, which has binding motifs in the *PLK4* promoter, as shown by Fan et al., who used the TRANSFAC database to study the interaction of KLF14 and *PLK4* in HeLa cells.^34^ Shen et al. suggested that ATF6 negatively regulates *PLK4* transcript by recruiting C/EBPβ to the upstream promoter of *PLK4* during endoplasmic reticulum (ER) stress, suggesting that ER stress may play a crucial role in *PLK4* expression.^38^ Moreover, p53 also represses *PLK4* through multiple mechanisms.^35^^,^^36^^,^^39^ The p53 activates the DREAM (dimerization partner, RB-like, E2F, and multi-vulval class B) complex, which binds CDE/CHR sites on the *PLK4* promoter^35^; interacts with DNA methyltransferase (DNMT)1 and DNMT3a to induce promoter hypermethylation 37, and recruits histone deacetylases (HDACs) to remove the acetyl group from the *PLK4* promoter, thereby suppressing *PLK4* transcription.^36^ *PLK4* mRNA has been shown to be repressed by non-coding microRNAs.^33^^,^^40^ The miR-126^33^ and miR-338-3p^40^ bind to distinct sites on the 3′ untranslated region (3′-UTR) of *PLK4* mRNA, destabilizing it and reducing *PLK4* expression (Figure 1C). These interactions have been observed in hepatocellular carcinoma (HCC)^33^ and neuroblastoma (NB)^40^ cell lines, highlighting the role of miRNAs in fine-tuning *PLK4* levels. Roberto et al. have shown that miR-10b∗ can alter *PLK4* expression in osteosarcoma cells. However, its exact mechanism to suppress *PLK4* remains unclear.^43^ Further, Phan et al. reported that upstream open reading frames located in the 5′-UTR of *PLK4* mRNA act as negative regulators of PLK4 translation initiation to prevent centriole amplification and preserve genomic integrity.^44^ Recently, BRD4, a bromodomain/extra-terminal family member, was found to be the epigenetic determinant of *PLK4* expression, shown to promote *PLK4* transcription in rat fibroblasts.^37^

The PLK4 protein also undergoes a tightly controlled auto-regulatory process. After protein synthesis, PLK4 exists in a monomeric, auto-inhibited state due to the linker 1 (L1) region, which blocks phosphorylation of the AL within the kinase domain, resulting in low kinase activity. PLK4 activation begins when CPB mediates its homodimerization, and the conserved PB3 relieves auto-inhibition by displacing L1 from AL. This dimerization is essential for initiating the kinase activity of PLK4. Subsequent phosphorylation events at different PLK4 sites further modulate various PLK4-mediated cellular processes.^45^ Phosphorylation of the AL fully activates the kinase, while phosphorylation of L1 promotes disassembly of the complex PLK4 protein structure, enhancing activity and enabling downstream processes.^23^

PLK4 self-regulates its degradation through auto-phosphorylation of a downstream regulatory element (DRE) within the L1 region. Phosphorylation of DRE at Ser285 and Thr289 are critical for initiating degradation, which recruits the Skp1-cullin-F-box complex (SCF)β-TRCP-E3 ubiquitin ligase complex.^46^ This in turn ubiquitinates PLK4 and processes it for proteasomal degradation. Interestingly, phosphorylation of Ser305 activates PLK4 but does not affect its stability.^45^ This active form (*p*-Ser305-PLK4) dynamically localizes during mitosis in different phases, from kinetochores to the midbody, suggesting a role in cell division.^23^ PLK4 protein is also regulated by other kinases and phosphatases, such as STIL,^47^ SAPKKKs,^48^ Asterless,^49^ TEC kinase 48, and PPA2/twins^50^ stabilize and positively regulate PLK4 activity. On the other hand, PIPKI-γ,^51^ FAM46C 51, and KAT2A/2B^52^ negatively regulate PLK4 activity (Figure 1C).

Ubiquitin modifications are also critical in PLK4 regulation. CYLD, a deubiquitinating enzyme, interacts with Spata2, an adaptor protein in ubiquitination pathways, and stabilizes PLK4 by removing the ubiquitin.^53^ This stabilized form phosphorylates NEK7, disrupting its interaction with NLRP3 and suppressing inflammasome activation.^53^ This is a PLK4-mediated regulatory pathway of NLRP3 inflammasome, which occurs at the centrosome and suggests a possible role of PLK4 in NLRP3-associated inflammatory diseases.^53^ Similarly, the E3 ligase activity of Mib1 ubiquitinates PLK4 at multiple sites, forming Lys11-, Lys29-, and Lys48-ubiquitin linkages. Lys11 and Lys48 promote proteasomal degradation of PLK4, whereas Lys29 impairs PLK4 interaction with CEP152 and CEP192, affecting centriole duplication.^54^ CAND1, a regulator of cullin-RING Ligases, further modulates PLK4 stability by binding CUL1 (cullin 1) and preventing SKP1 incorporation into the SCF complex. This was observed in prostate cancer cells, where CAND1 upregulation reduced ubiquitination and enhanced PLK4-mediated centriole amplification.^55^

### Cartwheel assembly and centriole duplication

**Figure 2:**
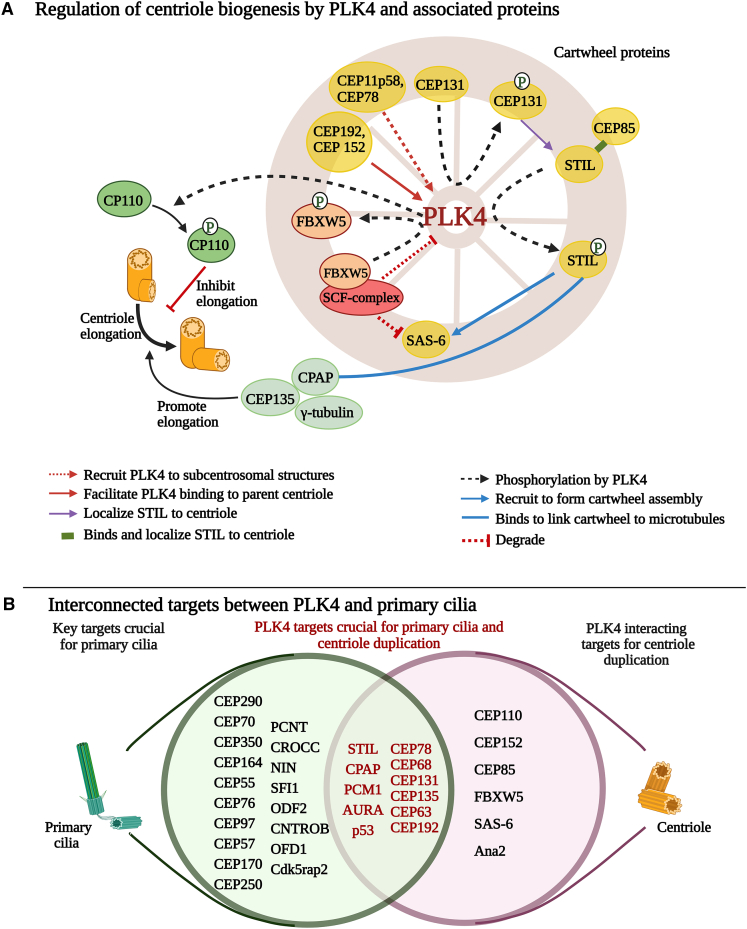
PLK4 interactions with proteins/target for centriole biogenesis and primary cilia (A) Cartwheel assembly proteins that regulate centriole biogenesis. CEP11p58 and CEP78 recruit PLK4 to subcentrosomal structures and CEP192 and CEP152 aids in binding PLK4 to parent centriole. CEP131, phosphorylated by PLK4, and CEP85 localize STIL to centriole and aids in binding to PLK4. PLK4-mediated phosphorylated STIL recruits SAS-6, which oligomerizes to form cartwheel assembly and stabilizes PLK4 for centriole biogenesis. Phosphorylated STIL binds with CPAP, which links cartwheel to microtubules wall. CPAP in conjunction with CEP135, and γ-tubulin, facilitates PLK4-mediated procentriole elongation. Overextension of centrioles is controlled by CP110 phosphorylation by PLK4 and degradation of PLK4/SAS-6 by SFC/FBXW5-complex. (B) Key proteins, which are crucial for formation and function of primary cilia adapted from Ho et al*.*^61^ The Venn diagram represents the proteins that are necessary for centriole duplication and/or primary cilia based on data from Ho et al*.*^61^ The overlapping circles of Venn diagram represent the proteins that interact with PLK4 and are crucial for both primary cilia and centriole duplication. Created in BioRender. Muntaqua, D. (2025) https://BioRender.com/hotsj52.

PLK4 localizes at the centrioles, where its concentration increases during the S phase and peaks at the G2/M phase.^29^ PLK4 regulates centrosome duplication to once per cell cycle, ensuring optimal cell division.^29^ Centrosomes, also known as the microtubule organizing center (MTOC), are organelles constructed by two centrioles (generated from microtubule triads) and surrounded by pericentriolar material (PCM).^56^ As the cell prepares for centrosome duplication, PLK4 is recruited to the end of the parent centriole, facilitated by PCM-dependent mechanism, which initiates cartwheel assembly involving multiple proteins, including SAS-6, centrosomal proteins (CEP192, CEP152, CEP135, and CEP78),^57^^,^^58^ STIL, and CPAP^23^ (Figure 2A). The CEP192 and CEP152 proteins interact with CPB, whereas CEP78 and CDK11p58 interact with the N-terminal catalytic domain of PLK4 to localize PLK4 to subcentrosomal structures and facilitate centriole duplication. Next, PLK4 phosphorylates CEP131 on T205 and S21, which then recruits STIL to the centriole. CEP85 aids STIL localization to centrioles.^59^ PLK4 further binds with STIL through its PB3 domain and phosphorylates the STAN motif on STIL. Subsequently, STIL recruits SAS-6, which oligomerizes to form centriolar cartwheel.^59^ The interaction of all these proteins pins down the area for procentriole biogenesis and constitutes the cartwheel assembly necessary for centrosome duplication. Additionally, STIL is phosphorylated at S428 by PLK4 and promotes STIL-CPAP binding. This links the cartwheel to microtubules of the centriole wall.^60^ The PLK4-STIL-SAS-6 complex stabilizes PLK4 around the mature centriole,^61^ whereas CPAP, CEP135, and γ-tubulin facilitate PLK4-mediated procentriole elongation^62^ (Figure 2A).

Lee et al. showed that PLK4 can control centriole elongation by phosphorylating CP110 on Ser98, which is located at the distal and partially at the proximal end of two centrioles.^63^ CP110 serves to limit the overextension of centrioles.^64^ It has also been reported that SCF-Slimb/β-TRCP-E3 ubiquitin ligase complex degrades PLK4 and SAS-6, which destabilize cartwheel assembly, halting the centriole duplication.^46^ Interstingly, Puklowski et al. suggested that PLK4 can phosphorylate and inactivate FBXW5, a component of the SCF complex, to inhibit the degradation of SAS-6. This, in turn, will stabilize cartwheel assembly and promote centriole duplication.^65^ Taken together, the precise functioning of the cartwheel proteins is essential for sustaining PLK4 activity and ensuring proper centriole duplication.

## PLK4 and genomic instability

A plethora of studies have suggested a crucial role of PLK4 in genomic stability.^56^^,^^66^^,^^67^^,^^68^^,^^69^^,^^70^ Dysregulated PLK4 activity can disrupt the normal function of cartwheel assembly and cause centriole overduplication, chromosome mis-segregation, and cytokinesis failure.^29^^,^^71^ These mitotic errors may induce genomic instability with aberrant chromosome copy numbers, cell cycle dysregulation, and dysfunctional mitotic checkpoints resulting in tumorigenesis.^1^ For instance, Kim et al. found that overexpression of CEP131 excessively recruited STIL, which stabilized the PLK4 and cartwheel assembly, causing centriole overduplication and progression of colon cancer.^72^ PLK4 overexpression was also associated with the dysregulation of the E2F transcription factor. E2F can bind between exon 1 and exon 2 of the *PLK4* promoter and stimulate transcription of *PLK4*, leading to genomic instability and the proliferation of the mammary epithelial cell line.^32^ The depletion of CEP78, which co-localizes with PLK4 in centrosomes, causes PLK4-induced overduplication of centrioles.^66^ PLK4-mediated phosphorylation of Ect2 and activation of RhoA GTPase are necessary for cytokinesis and to maintain the distribution of genomic material to progeny cells.^68^ In breast cancer cells, it was observed that PLK4-dependent RhoA activation promoted cell migration and invasion.^68^ Rosario et al. reported that haploid levels of PLK4 disrupt RhoA GTPase function during cytokinesis, resulting in aneuploidy and tumorigenesis.^69^ Kazazian et al. reported that PLK4 can phosphorylate and activate Arp2/3 complex, an actin cytoskeleton reorganizing complex, which is necessary for mitotic spindle organization and genomic stability.^10^ The Arp2/3 complex facilitates cell migration by creating new, branched actin filaments at the leading edge of a cell, which grow from existing filaments. This functional interaction between PLK4 and Arp2 was observed in PLK4-driven breast cancer cell motility via regulating Arp2/3-mediated actin cytoskeletal rearrangement.^10^ Liu et al. further showed that downregulation of PLK4, STIL, and CEP85 in osteosarcoma cells reduced Arp2-mediated actin reorganization and cancer cell migration.^47^ Thus, PLK4 plays an integral role in supporting centrosome activity, and its dysregulation triggers genomic instability that may result in cancer development and progression. An additional contributor to genomic instability includes primary cilia that is affected by PLK4 deregulation and is described in the next section.

## PLK4, primary cilia, and cancer

As described earlier, PLK4 plays a crucial role in centrosome duplication and dysregulation of centrosomes, whether in number or function, and is the leading cause of genomic instability. The mother centriole of the centrosomes serves as the basal body from which the primary cilium, a microtubule-based organelle, extends. There is one cilium present per cell, which extends from the apical surface of mammalian cells and has evolved to perform specialized sensory signaling functions.^73^^,^^74^ These sensory hubs are enriched in receptors and signaling pathways that facilitate important cellular processes such as development, growth, differentiation, migration, cell cycle regulation, and signal transduction.^75^ Their expression is tightly dependent on the cell cycle, with primary cilia present during G1/G0 phases where centrioles act as basal bodies that template the formation of primary cilia. These retract into the cell during mitosis when centriole duplication is under progress.^75^ This supports their crucial role in regulating cell division and maintaining epithelial phenotype.^74^ Hoh et al. explored the transcriptional profile of multi-ciliated tracheal epithelial cells to explore the gene expression during ciliogenesis. The transcriptional profile was subdivided into early and late time points during primary cilia assembly. A total of 649 genes were upregulated in the early category (as basal bodies are being formed), while 73 genes were upregulated in the late category (where most cells have cilia). Interestingly, *PLK4* was the most upregulated gene during basal body formation as compared to ciliated cells, suggesting a key role during early ciliogenesis.^61^ Additionally, multiple key ciliary proteins associated with the top differentially expressed genes, such as STIL, CEP192, p53 etc. are known to be phosphorylated by PLK4^61^ (Figure 2B). Mahjoub et al. demonstrated that PLK4 overexpression in mouse embryonic fibroblasts caused the formation of extra centrioles in the cells. Many of these extra centrioles formed a cilium, resulting in super-ciliated cells.^76^ This suggests that PLK4 may be linked to the formation of primary cilia. However, detailed research is needed to explore this potential connection.

A number of studies have demonstrated that cilia are absent from certain cancers, such as pancreatic and breast cancers, whereas others, such as small cell lung cancer, rely on cilia or ciliary signaling for their progression.^77^ Loss of primary cilia has been reported in multiple malignancies, such as melanoma, breast, thyroid, renal, liver, pancreatic, colorectal, and prostate cancers.^78^^,^^79^^,^^80^^,^^81^^,^^82^^,^^83^^,^^84^ The precise mechanism driving permanent cilium loss in cancers likely involves multiple factors, with PLK4 being a potential key player.^85^ Coelho et al. showed that PLK4 overexpression in *p53* null mice leads to hyperproliferation of epidermal cells, tumor development, and primary cilia loss in proliferating cells.^85^ This is contrary to the results reported by Mahjoub et al. as discussed earlier. This phenomenon may be linked to increased cell proliferation, as was suggested by Coelho et al. that repeated centriole duplication due to PLK4 overexpression prevents formation of basal bodies, implying that a high proliferative state may interfere with cilia formation.^85^ It is plausible that the impact of PLK4 overexpression on cilia formation may be context-dependent, varying with cell type and the proliferative state of the cells. Currently, there is no direct evidence linking PLK4-induced primary cilia loss to cancer and requires detailed investigation.

Important cancer pathways such as Notch, Sonic Hedgehog (Shh), mTOR, and Wnt are impacted by primary cilia signaling. These pathways play crucial roles in tumorigenesis, metastasis, and patient survival.^73^^,^^77^ Stasiulewicz et al. demonstrated that Notch1 causes pronounced accumulation of Smoothened (Smo) in primary cilia that leads to longer primary cilia in mouse/chick embryos and fibroblasts.^86^ Interestingly, primary cilia are retained in small cell lung cancer with activated Hedgehog signaling.^87^ In the context of pancreatic tumors, Bailey et al. found primary cilia on normal ductal cells of the pancreas, but did not observe primary cilia on the ductal tumor cells, which instead produced Shh. They observed localization of the receptor Smo along primary cilia in the stroma surrounding the tumor cells, suggesting the potential for Shh pathway activation in the local tumor microenvironment.^88^ In another study, aberrant primary cilia regulation has been linked to breast tumorigenesis. Overexpression of the centriolar protein SAS-6 enhances ciliogenesis in breast cancer cells and promotes invasion.^89^ SAS-6, a key partner of PLK4 in centriole duplication, is implicated alongside PLK4 in breast cancer.^89^^,^^90^

KIFC1 (kinesin family member C1), a motor protein crucial to the transport of membrane proteins from the Golgi to the primary cilium,^91^ has been shown to modulate PLK4 through TRIM-37. Zhou et al. demonstrated that overexpression of KIFC1 reduced PLK4 degradation through TRIM-37-mediated ubiquitination. This interaction promoted centrosome amplification and proliferation of endometrial cancer cells *in vitro.*^92^ Hori et al. showed that phosphorylation of PCM1 at S372 by PLK4 is critical to primary cilium formation.^67^ PCM1 supports primary ciliogenesis in glioblastoma, and its depletion increases susceptibility to temozolomide treatment, partly due to defective primary cilia formation.^93^ Later, PLK4 overexpression was reported to facilitate PCM1 aggregation and survival of osteosarcoma cells.^94^ Nonetheless, the extent to which PLK4 overexpression influences primary cilia loss or rescue in cancer remains undetermined. The interplay of primary cilia and PLK4, particularly in cancer signaling, seems an interesting field for future investigation.

## PLK4 in cancers

**Figure 3:**
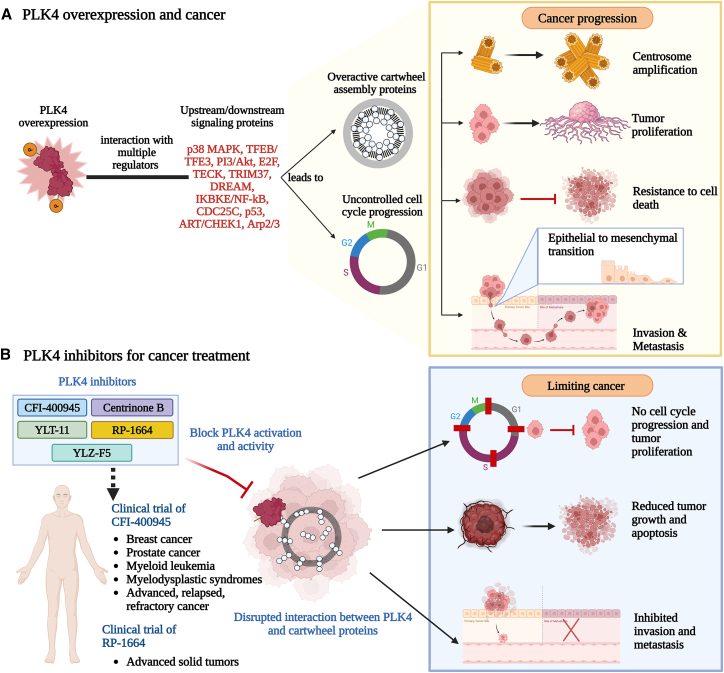
PLK4 as a target for cancer management (A) PLK4 overexpression either regulates or is regulated by multiple upstream and downstream proteins, which results in constant activity of cartwheel proteins to maintain PLK4’s active state and centriole overduplication (left panel). This in combination with uncontrolled cell cycle progression causes centrosome amplification and uncontrolled proliferation of cancer cells (right panel). Interaction of PLK4 with upstream and downstream signaling pathways provides resistance to apoptosis, helps cancer growth, and upregulates epithelial-mesenchymal transition, invasion, and metastasis (right panel). (B) Targeted therapy with small molecule PLK4 inhibitors blocks the PLK4 activity and disrupts the function of cartwheel proteins. This stops aberrant centrosome amplification and inhibits cell cycle progression and tumor proliferation. PLK4 inhibitors reduce tumor growth, induce apoptosis, and prevent tumor invasion and metastasis. PLK4 inhibitors, CFI-400945 and RP-1664 are under clinical trials for their anticancer activity in different cancers. Created in BioRender. Muntaqua, D. (2025) https://BioRender.com/k8xbovk.

Multiple investigations have been conducted to define the role and significance of PLK4 in cancer. PLK4 accumulates in actively dividing cells since it regulates the G2/M phases of the cell cycle.^23^ PLK4 is not an often-mutated gene in neoplasms; however, its overexpression has been associated with cancer, and it is being widely investigated as a therapeutic target for cancer management.^5^^,^^11^^,^^70^^,^^85^^,^^95^^,^^96^^,^^97^^,^^98^^,^^99^^,^^100^^,^^101^^,^^102^^,^^103^^,^^104^^,^^105^^,^^106^^,^^107^^,^^108^^,^^109^^,^^110^^,^^111^ Below, we have focused on studies showing the association of PLK4 with cancers *in vitro* and *in vivo* (Figure 3A). Additional highlights of these studies are provided in Table 1.

**Table 1:** Studies on the association between PLK4 upregulation and cancer

Cancer	Model	Outcomes/Features	References
Breast cancer	Patient samples	• Upregulated PLK4 mRNA expression levels • Poor relapse-free survival	Jiawei et al.^98^
	*In vitro c*ell line	• Upregulated PLK4 increased invasiveness of cells • siRNA PLK4 obstructed cell cycle checks and colony formation	Kahl et al.^99^
	*In vitro* cell line	• TRIM37-mediated cell-cycle arrest at the G2/S phase • 17q23/TRIM37 overexpression and PLK4 inhibition abridged mitotic spindle assembly and caused cell death	Yeow et al.^112^
	*In vitro* cell line *In vivo* xenograft model mouse model	• YLT-11 exhibited significant antiproliferation activities • Include apoptosis in cancer cells by inhibiting PLK4 • YLT-11 suppressed tumor growth	Lei et al.^101^
	*In vitro* cell line *Ex vivo* patient-derived organoid model	• Combination of CFI-400945 and radiation therapy • Synergistic anti-tumor effect	Parsyan et al.^16^
	*In vitro* cell lines	• PLK4 inhibition induced centrosome-centriole abnormalities in cells • PLk4 inhibitors suppressed proliferation and migration of cells	Sun et al.^113^
	Patient samples *In vitro* HER2-positive cell lines	• PLK4 overexpression • Trastuzumab resistance • Poor prognosis	Marina and Saavedra^114^
	*In vitro* cell lines	• PLK4 can mediate anoikis resistance • Induction of stable hybrid epithelial-mesenchymal phenotype	Fonseca et al.^115^
Cervical cancer	*In vitro* cell lines	• HPV E7 prompted overexpression of PLK4 • E7 inhibited DREAM and repressed the transcription of PLK4 in its promotor region	Fischer et al.^35^; Korzeniewski et al.^116^
Endometrial and uterine cancers	Patient sample *In vitro* cell lines *In vivo* xenograft mouse model	• PLK4 overexpression • CFI-400945 suppresses uterine cancer *both in vitro* and *in vivo* • PLK4 activity related to KIFC1 in endometrial cancer	Zhou et al.^92^; Lee et al.^100^; Zhao et al.^111^
Gastric adenocarcinoma	Patient samples	• Upregulation of PLK4	Guo et al.^97^
	*In vitro* cell line	• Upregulation of variant 1 of PLK4	Shinmura et al.^70^
Bladder cancer	*In vitro* cell lines *In vivo* xenograft model	• Inhibition of PLK4 by CFI-400945 and KO reduced cell proliferation and reduced tumor growth • PLK4 inhibition decreased cell proliferation • PLK5 inhibition enhanced phosphorylation of p38 MAPK and p53 and increased p21 and reduced expression of cyclin D1	Yang et al.^21^
Colorectal cancer	*In vitro* cell line *Ex vivo* cells (polyploid giant cells)	• Upregulation of PLK4 with higher expression of EMT-related proteins • PLK4 phosphorylating CDC25C affects the cell cycle	Fu et al.^96^
	*In vitro* cell line	• ROS-induced cleavage of ATF6 and elevation of PLK4	Bian et al.^6^
	*In vivo* xenograft mouse model	• CEP131 overexpression promoted PLK4-mediated cancer progression	Kim et al.^72^
	Patient samples *In vitro* cell lines	• PLK4 overexpression associated with poor survival • PLK4 knockdown increased chemo-sensitivity of cancer cells to 5-fluorouracil	Duan et al.^8^; Oh et al.^12^
Renal cell carcinoma	*In silico* analysis (TIMER, UALCAN, MethSurv, NCBI-GEO, and UCSC databases)	• Hypermethylation of PLK4 sites including cg22112850, cg06015521, and cg26882168	Hu et al.^117^
Acute myeloid leukemia	*In vitro* cell lines	• PLK4 inhibitor centrinone induced apoptosis and cell-cycle arrest at the G2/M phase	Mu et al.^11^
Multiple myeloma	*In vitro* cell lines	• CFI-400945 and bortezomib combination • Reduced PLK4 and cell viability • Amplified the sensitivity of MM cells to bortezomib therapy • Inactivated PI3K/Akt signaling	Xu et al.^118^
B-cell lymphoma	*In vitro cell* lines *In vivo* xenograft mouse model	• CFI-400945 blocked PLK4 • Delayed tumor progression with the activation of p53 and Hippo/YAP tumor suppressor signaling pathway	Zhao et al.^119^
Hepatocellular carcinoma	Patient samples, *In vitro* cell lines	• Overexpression of PLK4 in tissue samples • 2- to 3-fold faster migration and invasion of TetON PLK4 cells • TEC kinase can phosphorylate and stabilize PLK4 and promote HCC cell migration • Reduced proliferation and migration in KO	Yeung et al.^120^
	*In silico* analysis (GTEx and TCGA)	• A significant association between rs3811741 located in the PLK4 intron and HCC	Meng et al.^106^
	Patient samples *In vitro* cell lines	• Downregulation of mir-126 in tissues • Upregulation of PLK4 • Triggered the ATR/CHEK1 pathway	Bao et al.^33^
Lung cancer	*In silico* analysis (Oncomine database)	• Higher transcriptional levels of PLK4 in LSCC based on methylation status	Deng et al.^121^
	Patient samples	• Upregulation of PLK4 • Poor patient prognosis • Greater tumor size, metastasis, and higher TNM stage	Zhou et al.^122^
	*In vitro* cell lines	• CFI-400945-mediated downregulation of PLK4 • Apoptosis halting the proliferation of cancer	Kawakami et al.^9^
	Patient samples *In vitro* cell line	• Upregulated PLK4 and DNA Polymerase Theta (POLQ) • Significant polyploidy and promoted oncogenesis • Induced centrosome amplification	Shinmura et al.^123^
	*In vitro* cell line	• Reduced proliferation of TFEB and TFE3 double-knockout cells with PLK4 inhibitor-centric one	Kao et al.^124^
Glioblastoma	Patient samples *In vitro* cell lines *In vivo* intracranial xenograft mouse model	• Upregulation of PLK4 • Resistance to temozolomide and radiotherapy • PLK4 KO significantly increased the radio sensitivity of GBM cells • ATAD2-dependent transcriptional regulation of PLK4 promoted cell proliferation and radio-resistance	Wang et al.^42^
	*In vitro* cell lines *In vivo* patient-derived xenograft model	• PLK4 phosphorylated IKBKE causing elevation of NF-κB transcriptional activity and tumor proliferation • Temozolomide and CFI-400945 treatment slowed tumor growth • CFI-400945 treatment significantly restored temozolomide sensitivity	Zhang et al.^41^
	*In vitro* cell lines *In silico* analysis (GTEx/TCGA)	• EphA2 activation and proliferation, migration, and vasculogenic mimicry of glioma • Activated the PI3K-Akt and MAPK signaling	Wang et al.^125^; Wang et al.^126^
Medulloblastoma	*In vitro* cell lines *In vivo* xenograft mouse model	• Overexpression of PLK4 • CFI-400945 instigated polyploidy, DNA damage, and apoptosis	Sredni et al.^18^
Neuroblastoma	Patient samples	• Overexpression of PLK4 in primary and metastatic neuroblastoma	Bailey et al.^5^
	*In vitro* cell lines	• Overexpression of PLK4 and downregulation of tumor suppressor miR-338-3p • miR-338-3p can restrain the expression of PLK4. • shRNA PLK4 KO limit primary to the metastatic transformation of neuroblastoma	Tian et al.^20^; Zhang et al.^40^
Rhabdoid tumors	*In vitro* cell lines	• PLK4 inhibitor CFI-400945 or PLK4 KO reduced cell proliferation	Sredni et al.^127^
Osteosarcoma	*In silico (Gene Expression Omnibus database)* *In vitro* cell line	• PLK4 downregulation in senescent cells • PLK knockdown halted the proliferation of U2OS cells	Ledoux et al.^31^; Xie et al.^109^
Thyroid cancer	Patient samples *In vitro* cell lines	• Overexpression of PLK4 • Poor disease-free survival • Centrinone reduces cell proliferation and promotes apoptosis • Downregulation of Wnt/β-catenin pathway in cell lines in C643 and 8305c cells	Zhu and Xie^128^; Hu et al.^129^
Pancreatic cancer	*In vivo* patient-derived xenograft model	• Truncated PLK4 activity after CFI-400945 treatment • Reduced tumor growth	Lohse et al.^103^
Prostate cancer	Patient tissue microarrays *In vitro* cell lines	• PLK4 was overexpressed. • PLK4 inhibitors CFI-400945 and centrinone B repressed the proliferation and prompted senescence in PCa cells	Singh et al.^17^
	*In vitro* cell lines	• Fraxetin inhibited PLK4. • Reduced activity of PI3K/Akt signaling • Curbed the proliferation and migration/invasion of cells	Ma et al.^14^
	*In vitro* cell lines	• NL13 is a novel PLK4 inhibitor • Suppress cancer *in vitro* and *in vivo* via reduced Akt signaling	Qiao et al.^130^
Melanoma	Patient tissue microarray	• Overexpression of PLK4 in melanomas versus benign nevi • Poor patient prognosis	Denu et al.^131^
	*In vitro* cell lines	• Centrinone B inhibition of PLK4 depleted centrioles and hindered the proliferation of melanoma cells	Denu et al.^131^
	Patient samples	• PLK4 overexpression correlated with lymph node metastasis, increased TNM stage, and poor survival	Zhang et al.^132^
Non-melanoma skin cancers	*In vivo* genetically modified mouse model	• PLK4 overexpression resulted in supernumerary centrosomes • Accelerated spontaneous skin tumors in adult epidermis	Serçin et al.^133^
	*In vitro* cell lines *In vivo* xenograft mouse model	• PLK4 overexpressed in basal and squamous cell carcinoma cells • CFI-400945 decreased proliferation and induced apoptosis in cancer cells • PLK4 knockdown inhibited tumor growth	Ndiaye et al.^134^
	*In vivo* genetically modified mouse model	• Epidermal thickening, progressive hair loss • Formation of spontaneous squamous cell carcinoma in the skin tissues • Centrosome amplification with elevated PLK4 levels	Levine et al.^102^

### Endocrine cancers

#### Thyroid cancer

Zhu et al. have demonstrated an overexpression of PLK4 in anaplastic thyroid cancer cell lines and inhibition of PLK4 resulted in decreased cell viability, induction of apoptosis, G2/M phase cell-cycle arrest, and deactivated Wnt/β-catenin pathway in C643 and 8305C cells.^128^ Recently, the importance of PLK4 as a biomarker in papillary thyroid carcinoma was assessed. PLK4 was overexpressed in tumor tissues and was linked with poor disease-free survival. It was also associated with extrathyroidal invasion and higher tumor stage in thyroid cancer.^129^

### Digestive cancers

#### Gastric and colorectal cancers

Multiple studies have provided evidence on the involvement of PLK4 in gastrointestinal cancers.^70^^,^^95^^,^^96^^,^^97^^,^^104^^,^^110^ PLK4 overexpression was associated with increased tumor, nodes, and metastasis (TNM) stage and shorter disease-free survival in stomach adenocarcinoma and colorectal cancer patients.^70^^,^^95^^,^^97^^,^^104^^,^^135^ Higher levels of PLK4 levels were also correlated with poor response to neoadjuvant chemo-radiation in rectal cancer patients who received preoperative chemo-radiation therapy.^12^ PLK4 overexpression upregulated epithelial-to-mesenchymal transition (EMT) markers in polyploid giant cancer cells, which declined upon PLK4 knockout in colorectal cancer.^96^ This was consistent with Liu et al. who showed that PLK4 can promote the formation of polyploid giant cells by interacting with and phosphorylating CDC25C, resulting in enhanced cell proliferation, invasion, and migration.^136^ One of the promoters of EMT is Wnt/β-catenin signaling, where the activation of Wnt can increase β-catenin accumulation in the cytoplasm, which in turn stimulates EMT and related genes upon nuclear translocation. The Wnt/β-catenin/EMT axis has been implicated in cancer metastasis.^137^ Liao et al. demonstrated that PLK4 knockdown results in the inactivation of Wnt/β-catenin pathways in colorectal cancer models.^135^ Duan et al. found that PLK4 knockdown decreased nuclear translocation of β-catenin and increased the chemosensitivity of colorectal cancer cells to 5-fluorouracil.^8^ Some of the heavy metals are classified as carcinogens as these can stimulate the progression of cancer and reduce patients’ sensitivity to treatment.^138^ Metals such as hexavalent chromium^6^ and cadmium^139^ prompt PLK4-mediated centrosome amplification in HCT116 colon cancer. This highlights PLK4 as a possible mechanism of heavy metal-mediated cancer and provides a therapeutic target for future studies. In short, the earlier mentioned studies indicate that PLK4 overexpression is correlated with gastrointestinal cancer. However, the molecular mechanism of PLK4 in these cancers needs further investigation.

#### Hepatic cancer

Interestingly, there are reports of both the upregulation and downregulation of PLK4 in hepatic cancer. Some studies have shown that lower levels of PLK4 were associated with faster tumor growth, advanced cancer stage, and poor patient survival.^69^^,^^140^^,^^141^ This was attributed to the loss of heterozygosity^69^^,^^141^ and hypermethylation of the *PLK4* promoter^142^ found in hepatocellular carcinoma tissues. On the contrary, overexpression of PLK4 was also shown to be associated with poor prognosis in hepatic cancer patients.^33^^,^^106^ Meng et al. found that the rs3811741 genetic variant located in the intron of the *PLK4* gene was considerably linked with HCC risk.^106^ This demonstrated that a particular single-nucleotide polymorphism in *PLK4* gene could alter susceptibility to cancer. The risk allele A of rs3811741 was associated with increased *PLK4* expression in liver cancer tissues, implying that it may be an oncogenic event. However, this requires further investigation. Yeung et al. have reported an interaction between PLK4 and TEC tyrosine kinase, which can phosphorylate and stabilize PLK4 and promote invasion, migration, and metastasis of HCC.^120^ In addition, PLK4 inhibition by CFI-400945 suppressed HCC growth in the *p53/PTEN* knockout mouse model.^7^ Bao et al. reported upregulation of PLK4 and downregulation of miR-126 in HCC tissues, which binds to the 3′-UTR of *PLK4* mRNA and represses its transcription. They reported significant downregulation of PLK4 in miR-126 overexpressing HCC cell lines. In addition, the PLK4/ATR/CHEK1 axis was suppressed in xenograft tumors grown from HCC cells overexpressing miR-126.^33^ This indicated that miR-126 was negatively whereas the ATR/CHEK1 axis was positively correlated with the oncogenic function of PLK4 in HCC cells.^33^ Taken together, there are different mechanisms by which both higher and lower expression of PLK4 was shown to be associated with progression and poor prognosis of HCC. It would be interesting to delineate the exact mechanisms and conditions where PLK4 inhibition or overexpression would be beneficial for the management of HCC.

#### Pancreatic cancer

Overexpression of PLK4 and centriole overduplication are recognized as markers of poor prognosis in aggressive pancreatic cancer.^85^^,^^103^ Coelho et al. observed hyperplasia in pancreatic islets in *p53* null mice with PLK4 overexpression.^85^ Furthermore, patient-derived xenografts (PDXs) of pancreatic cancer showed higher expression of *PLK4* mRNA along with *p53* mutations. Treatment of PDXs with a PLK4 inhibitor CFI-400945, reduced tumor growth, proliferation markers, and tumor-initiating cells.^103^ These limited number of studies provide evidence of PLK4’s association with pancreatic cancer. Additional detailed studies are needed to define the role and functional significance of PLK4 in pancreatic cancer.

### Genitourinary cancers

#### Bladder and renal cancers

Hu et al. performed *in silico* analysis of the methylation status of PLK4 in renal cell carcinoma.^117^ *PLK4* mRNA expression showed a significant negative correlation with its methylation levels, which were markedly lower in cancer tissues compared to normal tissues. In general, hypermethylation of *PLK4* represses its transcription levels. This suggested that high PLK4 expression in renal cell carcinoma may be regulated by *PLK4* methylation levels.^117^ It was further found that three sites of *PLK4*, including cg22112850, cg06015521, and cg26882168, were highly methylated, and the higher the methylation level, the better it was for cancer prognosis.^117^ Additionally, high *PLK4* mRNA expression was associated with higher proliferation of bladder cancer cell lines. PLK4 knockdown or inhibition using CFI-400945 showed decreased cell proliferation, enhanced phosphorylation of p38 MAPK and p53, increased p21, and reduced expression of cyclin D1.^21^ Similar results were observed in xenograft model showing reduced tumor growth as compared to the control group.^21^ These studies indicate that PLK4 dysregulation is correlated with bladder and renal cancers, which warrant detailed investigations.

#### Prostate cancer

Recently, our laboratory reported that PLK4 overexpression induced centrosome amplification in prostate cancer tissues as compared to prostatic hyperplasia or benign prostate tissues. PLK4 was also associated with poorer disease-free survival.^17^ In addition, treatment with PLK4 inhibitors CFI-400945 and centrinone B reduced migration, invasion, and proliferation of both androgen-responsive and androgen-independent prostate cancer cells.^17^ Another study on prostate cancer cells showed that PLK4 expression was reduced upon treatment with a natural product called Fraxetin, which suppressed the proliferation, migration, and invasion of cells. These effects were reversed by PLK4 overexpression.^14^

#### Testicular cancer

Zhang et al. investigated the hub genes associated with testicular germ cell tumor (TGCT) by analyzing datasets from the Gene Expression Omnibus using a bioinformatics approach.^143^ Their findings were validated using data from The Cancer Genome Atlas, the Genotype-Tissue Expression database, and the Human Protein Atlas. The authors observed a downregulation of *PLK4* in TGCT, while its expression was shown to remain relatively high in normal testicular tissue. The authors suggested that *PLK4* mRNA could serve as a potential diagnostic marker for TGCT and may play a unique role in its pathogenesis.^143^ The elevated *PLK4* levels in testicular tissue might be attributed to spermatogonia, which are actively involved in spermatogenesis and capable of continuous proliferation. In addition, analysis of the relationship between hub genes and immune cell infiltration showed that *PLK4* expression had a positive correlation with the infiltration of central memory T cells (Tcm) and Th2 cells. In contrast, it was negatively correlated with a broad range of immune cells, including activated dendritic cells (aDCs), follicular helper T (TFH) cells, Th17 cells, B cells, various subsets of natural killer (NK) cells, regulatory T (TReg) cells, cytotoxic cells, eosinophils, CD8^+^ T cells, neutrophils, mast cells, Th1 cells, macrophages, and multiple dendritic cell types (DC, pDC, and iDC).^143^ These results require validation using different testicular cancer models.

Additional studies on the interaction of PLK4 with molecular pathways in genitourinary cancers would offer deeper insight into their pathogenesis and give more therapeutic options for their management.

### Gynecological cancers

#### Breast cancer

Breast cancer is a significant health concern for females, where PLK4 has been shown to be dysregulated. Based on several studies, PLK4 overexpression has been associated with poor relapse-free survival^98^^,^^99^^,^^101^^,^^105^ and resistance to trastuzumab and tamoxifen therapy,^114^ in breast cancer patients. It was demonstrated that centrosome amplification elicited by PLK4 increased the invasiveness of breast cancer.^99^ Additionally, PLK4 was found to be a negative predictor of response to taxane-based neoadjuvant chemotherapy, indicating its potential for personalized medicines to help tailor treatment to individual patients’ needs.^90^ It was also reported that the treatment of breast cancer cells that overexpressed *17q23/TRIM37* with centrinone hindered the mitotic spindle assembly and accelerated cell death.^105^ PLK4 overexpression was correlated with dysregulation of transcription factors E2F activators^32^ and a regulatory kinase of the centrosome, NEK2.^114^ Recently, PLK4 has been shown to mediate anoikis resistance in breast and mammary epithelial cells with the induction of a stable hybrid epithelial-mesenchymal phenotype.^115^ These results support the use of PLK4-targeted therapies in breast cancer.

#### Cervical cancer

Cervical cancer is caused mostly by human papillomavirus (HPV) oncoproteins E6 and E7,^144^ which can dysregulate mitosis and induce genomic instability. It was demonstrated that HPV E7 oncoprotein requires PLK4 to promote centriole overduplication. Downregulation of PLK4 with siRNA impaired the ability of HPV E7 to induce centriole overduplication in cancer cells.^30^ In addition, HPV E7 can inhibit DREAM-CDE/CHR pathway in the promoter region of *PLK4* and upregulate *PLK4* transcription.^30^ This leads to PLK4-mediated centrosome amplification and proliferation of cervical cancer.^30^^,^^35^

#### Endometrial and uterine cancers

Recent studies have shown the association of PLK4 with endometrial and uterine cancers. Genomic profiling of clinical uterine leiomyosarcoma showed overexpression of PLK4. The PLK4 inhibitor CFI-400945 demonstrated significant antitumor activity in SK-UT-1, SKN, and SK-LMS-1 and SK-UT-1 cell lines and uterine cancer xenografts.^100^ Further, Zhao et al. demonstrated that higher PLK4 protein expression was positively associated with cancer progression and shortened survival in endometrial cancer patients.^111^ Interestingly, PLK4 activity was correlated to KIFC1, where KIFC1 overexpression promoted the expression of PLK4 and centrosome amplification in endometrial cancer HEC-1A cells, suggesting the importance of PLK4 in the progression of endometrial cancer.^92^

In short, PLK4 may have a role in the progression of breast, uterine, and cervical cancer, whose mechanism is not established yet. Further investigations are imperative for a better understanding of the extent of PLK4 involvement, its underlying biological mechanisms, and identification of novel treatment strategies.

### Hematological cancers

Recently, Mu et al. revealed higher PLK4 levels in acute myeloid leukemia (AML) cells, where PLK4 knockdown or inhibition by centrinone caused apoptosis and induced cell-cycle arrest at the G2/M phase with a decrease in expression of STAT3.^11^ Xu et al. established PLK4 overexpression in multiple myeloma cells, which, when blocked, reduced cell viability, induced apoptosis, and amplified the sensitivity of multiple myeloma cells to chemotherapy.^118^ In another study, the authors found an association of PLK4 overexpression with diffuse large B-cell lymphoma. PLK4 inhibition suppressed *in vitro* cell proliferation and *in vivo* tumor progression with the activation of p53 and Hippo/YAP signaling pathway.^119^ These studies provide evidence that PLK4 contributes to the growth of hematologic cancers. However, its exact mechanism in these cancers is still unknown.

### Integumentary and musculoskeletal cancers

#### Osteosarcoma

A few studies have found an overexpression of PLK4 in osteosarcoma.^108^^,^^109^ Bioinformatics analyses performed on osteosarcoma cells in quiescence and senescence stages showed that PLK4 was downregulated as compared to actively proliferating cells^109^ indicating its influence on the cell cycle in osteosarcoma. An *in vitro* study has shown that depleting PLK4 using siRNA led to a reduction in cell proliferation and decreased the number of centrioles in osteosarcoma cells.^31^ This was attributed to NF-κB, which directly targets PLK4 and plays a crucial role in osteosarcoma cell proliferation, an important regulatory pathway which is involved in a variety of cellular processes including inflammatory and immune responses, cellular death, stress, adhesion and progression. Depletion of NF-κB in osteosarcoma cells reduced the PLK4 mRNA and protein levels and resulted in defects in centrosome structure.^31^ It is plausible that regulation of PLK4 by NF-κB could be a key pathway that contributes to genomic instability in cancer, either driven by inflammation or oncogenic signals. In another study, oxygen deprivation of osteosarcoma cells was found to cause hypermethylation in the *PLK4* promoter region, decreased *PLK4* transcription, and reduced cell proliferation.^39^ These findings suggest a role of PLK4 in osteosarcoma. However, additional studies are required to determine the exact role of PLK4 in the pathogenesis of osteosarcoma.

#### Skin cancer

The role of PLK4 in skin cancers is beginning to be investigated. Some studies have shown that PLK4 overexpression triggers changes in skin tissues that are prerequisites for cancer development.^85^^,^^102^^,^^107^^,^^133^ Coelho and colleagues observed hyperproliferation of skin cells, loss of differentiated melanocytes, thickening of epidermis, and increased expression of keratin 5 and 6 in a PLK4 overexpressing transgenic mouse model.^85^ Later, Sercin et al. reported that PLK4 overexpression accelerated skin tumors in mice but required concomitant p53 deletion.^133^ Levine et al. also found that mice overexpressing PLK4 showed centrosome amplification, epidermal thickening, follicular damage, progressive hair loss, and formation of tumors in the skin tissues.^102^

Studies from our laboratory and by others have assessed the role of PLK4 in melanoma and non-melanoma skin cancer (NMSC).^131^^,^^132^^,^^134^ PLK4 was found to be significantly overexpressed in melanoma tissues as compared to benign tissue, which correlated with poor patient survival. It was also validated in a panel of human melanoma cell lines, where PLK4 overexpression was correlated with centriole overduplication, and its inhibition by a centrinone B resulted in depleted centrioles and reduced proliferation of melanoma cells.^131^ Recently, Zhang et al. have found PLK4 overexpression in cutaneous melanoma patients undergoing surgical resection positively correlates PLK4 with lymph node metastasis, increased TNM stage, and poor survival in melanoma patients.^132^ Our laboratory reported PLK4 to be significantly overexpressed in basal cell carcinoma (BCC) and cutaneous squamous cell carcinoma (cSCC) cells and tissue samples. Inhibition of PLK4 using CFI-400945 and centrinone and genetic manipulation has shown significant growth inhibitory effects *in vitro* in cSCC and BCC cells and reduced tumorigenesis in a mouse xenograft model.^134^ This presented PLK4 as a crucial promoter of melanoma and NMSC progression, which requires further in-depth exploration using different *in vitro* and *in vivo* melanoma models.

### Nervous system tumors

#### Neuroblastoma, rhabdoid tumors, and medulloblastoma

Nervous system tumors such as neuroblastoma, rhabdoid tumors (RTs), and medulloblastoma (MB) are high-grade and aggressive tumors in children. Bailey et al. reported an overall poor prognosis with PLK4 overexpression in primary and metastatic NB.^5^ Another study found that PLK4 overexpression in NB tissues was negatively correlated with miR-338-3p, a tumor suppressor.^40^ *PLK4* is the direct target of miR-338-3p, which binds to 3′-UTR of *PLK4* mRNA and represses its transcription. Further, PLK4 activity was related to SNHG1/miR-338-3p axis in NB, where SNHG1 downregulated miR-338-3p and upregulated PLK4 to promote proliferation, migration, and invasion of NB cells.^40^ Similarly, Tian et al. demonstrated inhibition of the metastatic transformation of NB cells with PLK4 knockdown.^20^ A few studies have shown PLK4 overexpression in RT and MB.^5^^,^^18^^,^^127^ Sredni et al. found that PLK4 supports the proliferation of MB and RT cells, whereas PLK4 inhibition causes DNA damage and apoptosis in a xenograft model for MB.^18^ It was also demonstrated that PLK4 knockdown in rhabdoid cells inhibited their proliferation and survival.^127^

#### Glioblastoma

PLK4 overexpression was shown to be correlated with poor prognosis in glioblastoma patients.^42^ Wang et al. suggested that ATPase family protein ATAD2, upregulated PLK4 expression in GB cells,^42^ which prompted tumorigenesis and resistance to temozolomide and radiotherapy in GB cells.^42^ Another study showed that PLK4 induced the activation of IKBKE/NF-κB, which resulted in augmented tumor growth and chemoresistance in a mouse model.^41^ Recently, it was observed that PLK4 can significantly enhance EphA2 signal transduction and accelerate malignant transformation and vasculogenic mimicry in glioma cells.^125^ PLK4 knockout in glioma cells was shown to be correlated with differential regulation of focal adhesion pathway, MAPK signaling pathway, and PI3K/Akt signaling in a study by Wang and colleagues.^125^ Additionally, this group also demonstrated that PLK4 was involved in governing metabolic processes by activating PI3K/Akt/mTOR pathway in glioma cells.^126^ They identified Akt1 as a substrate of PLK4, which promoted glioma cell proliferation and invasion.^126^

Hence, targeting PLK4 can be beneficial for the management of nervous system tumors, subject to additional detailed studies in this area in human-relevant cancer models.

### Respiratory system cancer

Poor prognosis in lung cancer patients has been linked with overexpression of PLK4.^121^^,^^122^ Global methylation survival analysis using bioinformatics showed the prognostic value of PLK4 methylation in lung cancer.^121^ It was reported that a higher methylation level of *PLK4* was significantly related to the favorable survival probability of patients with lung adenocarcinoma.^121^ PLK4 was also associated with greater tumor size, metastasis, and higher TNM stage in lung cancers.^122^ PLK4 knockdown in lung cancer cells induced polyploidy and halted cell proliferation.^9^ Another study showed that DNA polymerase theta (POLQ) overexpression was positively correlated with PLK4 overexpression in lung adenocarcinoma and induced PLK4-mediated centrosome amplification in lung cancer cells. POLQ regulates DNA synthesis and DNA double-strand break (DSB) repair. Co-expression of PLK4 and POLQ caused polyploidy where POLQ may have contributed to resistance to DSBs and promoted cancer progression in lung adenocarcinoma.^123^ Kao et al. studied the role of transcription factor EB and E3 (TFEB/TFE3), the substrates of PLK4, and how they affect proliferation of lung cancer cells. In general, TFEB and TFE3 sustain lysosomal biogenesis and autophagy induction. PLK4 localizes TFEB and TFE3 in the cytoplasm by phosphorylating these at Ser459 and Ser560, respectively, preventing autophagy and lysosomal biogenesis. Inhibition of PLK4 by centrinone dephosphorylates and translocates TFEB and TFE3 to the nucleus and supports cancer proliferation in the absence of centrosome, implying there are impediments to adopting centrosome depletion alone as a treatment strategy. It was found that the inhibition of PLK4 in TFEB and TFE3 double-knockout cells drastically reduced the proliferation of lung cancer cells, providing a rationale for combination therapies.^124^ It will be important to study PLK4 in different models of lung cancer to get a better understanding of its precise role.

Taken together, the aforementioned studies have provided evidence of the role of PLK4 in multiple cancers. There is limited information on the molecular pathways associated with PLK4 in cancer pathogenesis. Further studies are needed to provide advanced insight into the molecular mechanisms of PLK4 in cancers. Interestingly, several researchers have assessed different PLK4 inhibitors in preclinical models, two of which are also used in clinical trials. In the next section, we have summarized available PLK4 inhibitors studied in regulating cancer growth.

## PLK4 inhibitors for cancer management

As discussed in the previous section, PLK4 has emerged as a promising therapeutic target for cancer management. Aberrant PLK4 expression has been shown to be frequently associated with tumorigenesis, making it an attractive candidate for targeted cancer therapy. Recent advances in the development of PLK4 inhibitors have opened new avenues for cancer management, particularly in malignancies characterized by high levels of chromosomal instability and/or PLK4 overexpression. Below, we have discussed published literature on small-molecule inhibitors of PLK4 in cancer management (Figure 3B).

### CFI-400945

CFI-400945, an indolinone derivative, is a selective PLK4 inhibitor at the IC_50_ of 2.8 nM.^15^ It is the only orally active ATP-competitive kinase inhibitor that is under clinical trials. Multiple preclinical studies have demonstrated significant anticancer activity of CFI-400945 as monotherapy as well as combination therapy with other anticancer drugs. CFI-400945 has shown promising anticancer results in colon, hepatic, breast, lung, and brain tumors.^7^^,^^9^^,^^16^^,^^18^^,^^96^^,^^145^ It caused mitotic cell death in MDA-MB-468 and MDA-MB-231 cells and repressed MDA-MB-468 tumor growth in the xenograft mouse model.^15^ It reduced the invasion, migration, and proliferation abilities of polyploid giant cancer cells *in vitro* in colorectal cancer model.^96^ Additionally, treatment of BC5637 and MGHU3 bladder cancer cells with CFI-400945 decreased the expression of cyclin D1, phosphorylated p38 MAPK, and p53 and increased the apoptosis marker p21.^21^ The HCT116 xenograft model of colon cancer and the *p53/PTEN* KO hepatocellular carcinoma mouse model are no exceptions, where tumor growth was suppressed *in vivo.*^7^^,^^145^ CFI-400945 delayed hepatic tumor growth *TP53*^*KO*^*/Myc*^*OE*^ or *PTEN*^*KO*^*/Myc*^*OE*^ mice by recruiting immune cells, including CD4^+^/CD8^+^ T cells, natural killer cells, and macrophages. Combination therapy of CFI-400945 with anti-PD1/PD-L1 antibody exhibited a propensity to improve overall survival.^7^ Kawakami et al. treated lung cancer cells and syngeneic lung cancer xenograft mice with CFI-400945. They found that CFI-400945 downregulated PLK4 and caused polyploidy, mitotic defects, and growth inhibition in both models.^9^ Similar results were obtained in prostate cancer, pancreatic cancer, pediatric brain tumors, neuroblastoma, RTs, and MB models.^17^^,^^18^^,^^103^^,^^127^ These studies provide evidence that CFI-400945 is a viable PLK4 inhibitor that has the potential as an effective anticancer drug.

The therapeutic potential of CFI-400945 has also been established in combination therapy regimens in preclinical studies.^15^^,^^16^^,^^118^^,^^119^ The treatment of the TNBC cell lines and patient-derived organoids with the combination of CFI-400945 and radiation therapy showed a synergistic antitumor effect as compared to radiation-only treatment.^16^ Zhang et al. reported that a combination of temozolomide and CFI-400945 restored temozolomide sensitivity, reduced the tumor burden, and improved the survival in the glioblastoma PDX model of the experimental animals.^41^ Xu et al. demonstrated the synergistic effect of CFI-400945 and bortezomib therapy in multiple myeloma cells.^118^ Similar results were obtained in the case of diffuse large B-cell lymphoma, where CFI-400945 in combination with doxorubicin significantly inhibited the proliferation of cells *in vitro* and markedly delayed tumor progression in the xenograft model.^119^ Collectively, CFI-400945 revealed strong anticancer activity in preclinical studies with marked tumor regression *in vitro* and *in vivo*. The combination of CFI-400945 with other therapeutic agents could provide great benefits for cancer therapy.

#### Clinical trials using CFI-400945

Multiple clinical trials are underway to assess the clinical efficacy and tolerability of CFI-400945 (Figure 3B). Initially, safety, tolerability, and pharmacokinetic profile were established in a phase 1 dose-escalation study (NCT01954316), which showed that CFI-400945 is well tolerated^146^ and the 64 mg dose was defined as the recommended phase 2 dose (RP2D).^147^ In another clinical investigation, a combination of CFI-400945 (32 mg/day; oral) with a PD-L1 inhibitor, durvalumab, is being assessed in patients with refractory triple-negative breast cancer in a phase 2 clinical trial (NCT04176848). Another phase 2 clinical trial is investigating CFI-400945 (32 mg/day; oral) in metastatic/advanced breast cancer (NCT03624543). CFI-400945 (64–224 mg/day; oral) was previously investigated in patients with relapsed or refractory acute myeloid leukemia and myelodysplastic syndrome (MDS) (NCT03187288). Data from nine patients was used to assess the effectiveness of the treatment, thus called efficacy evaluable. It was shown that three out of nine efficacy evaluable AML patients and two out of four patients with *TP53* mutations achieved complete remission.^148^ It is followed by another ongoing clinical trial (NCT04730258) that assesses the efficacy of the newer crystal form of CFI-400945 (32 mg/day; oral) with or without azacitidine or decitabine in AML, MDS, and chronic myelomonocytic leukemia. FDA has granted Fast Track status to CFI-400945 according to Treadwell Therapeutics for the treatment of adult individuals who have relapsed or refractory AML.^112^

Expanded clinical studies are required to confirm the safety and efficacy of CFI-400945 in larger patient populations and other types of advanced cancers. Further, clinical trials are also needed to show long-term benefits such as sustained tumor control, improved progression-free survival, and overall survival.

### Centrinone/centrinone B

Centrinone and centrinone B are structurally related compounds with a 1-piperidinyl ring at position 6 in centrinone B instead of a 4-morpholinyl ring in centrinone. These are selective PLK4 inhibitors that exhibit >1,000-fold selectivity for PLK4 over Aurora A/B *in vitro*^149^*.* Centrinone/centrinone B has also shown efficacy in lung, leukemia, thyroid, prostate, breast, and skin cancers.^11^^,^^17^^,^^112^^,^^120^^,^^124^^,^^128^^,^^131^^,^^150^ However, these compounds are not orally active and still have not assesses in clinical trials. Yet, these inhibitors have played a significant role in elucidating the role of PLK4 in cancers. We have shown that centrinone B repressed proliferation and prompted senescence in the prostate cancer cells. It inhibited cell growth, viability, and colony formation of both androgen-responsive and androgen-independent prostate cancer cell lines.^17^ In a previous study from our laboratory, centrinone B treatment in A375 and Hs294T melanoma cells, was found to deplete centrioles and hinder the proliferation of the melanoma cells.^131^ Indeed, minor chemical modification may improve the oral bioavailability and tolerability of these PLK4 inhibitors without affecting their efficacy profile. In fact, recently, chemical modifications based on structure-based drug design by Repare Therapeutics have resulted in bioavailable form of centrinone B, known as RP-1664. This compound is currently under phase 1 clinical trials to identify a safe and tolerated dose and examine the pharmacokinetics, pharmacodynamics, and preliminary anti-tumor activity in advanced solid tumors (NCT06232408). RP-1664 was shown to be effective in neuroblastoma models with activity against *TRIM37*-amplified, *TP53*-WT tumors. RP-1664 suppressed neuroblastoma both *in vitro* and *in vivo.*^151^

### Other compounds

YLT-11 and YLZ-F5 are the two (*E*)-4-(3-arylvinyl-1*H*-indazol-6-yl)pyrimidin-2-amine derivatives, which prevent PLK4 auto-phosphorylation. These compounds have been studied in breast and ovarian cancers.^22^^,^^101^ They have been shown to inhibit cell proliferation and migration, and induce mitotic defects and apoptosis.^22^^,^^101^ Recently, Qiao et al. have reported that NL13, a novel curcumin analog, is a potent PLK4 inhibitor that imparts significant anti-proliferative effects against prostate cancer in both *in vitro* and *in vivo* models. These effects were shown to be mediated by inactivation of the Akt signaling pathway after treatment with NL13, with decreased *p*-Akt and increased cleaved caspase-9/3.^130^ Wang et al. identified Akt1 as a substrate of PLK4, showing that PLK4 phosphorylates Akt1 at three distinct sites, S124, T308, and S473, thereby promoting glioma cell proliferation and invasion.^126^ Additionally, Tian et al. demonstrated that genetic inhibition of PLK4 suppressed neuroblastoma metastasis by downregulating the PI3K/Akt signaling pathway.^20^ These findings suggest a convergence between PLK4 activity and PI3K/Akt signaling in regulating the cell cycle^123^ and NL13 could be beneficial in treating cancer by targeting these pathways.

Another study demonstrated that novel pyrazole (3,4-day) pyrimidine derivatives significantly inhibited PLK4 kinase activity.^113^ The compounds exhibited notable antiproliferative activities against TRIM37-amplified breast cancer cells by inhibiting PLK4.^113^ TRIM37 modulates mitotic machinery and the function of centrosome proteins.^152^ Its amplification ensues in about 10% of cases of breast cancer, where it results in mitotic catastrophe. It was previously reported that the deletion of PLK4 results in TRIM37-mediated cell-cycle arrest.^153^ It was shown that treatment of breast cancer cells that overexpressed *17q23/TRIM37* with PLK4 inhibitor hindered the mitotic spindle assembly and accelerated cell death. Similar results were obtained in patient-derived organoids presenting PLK4 as a target of therapy in TRIM37-amplified cases of breast cancer.^105^ Sun et al. observed cell-cycle arrest at G2/S phase in MCF-7 cells after the inhibition of PLK4 by a compound called 1-(3-fluoro-5-(5-(3-(methylsulfonyl)phenyl)-1H-pyrazolo[3,4-*b*]pyridin-3-yl)phenyl)-3-(pyrimidin-5-yl)urea.^113^^,^^154^

Taken together, PLK4 inhibitors are currently being evaluated in both preclinical and clinical settings, showing potential for cancer management. These inhibitors may offer therapeutic benefits for patients with progressive disease who have not responded to standard therapies, potentially improving overall survival outcomes. Notably, the fast-track designation and encouraging preclinical results of CFI-400945 highlight the promise of PLK4 inhibitors as next-generation cancer therapeutics. Furthermore, high-throughput screening approaches could facilitate the identification of novel targets and combination strategies to enhance the efficacy of PLK4-targeted therapies.

## PLK4 signaling: *Future perspectives*

PLK4 has been recognized as a master regulator of centriole duplication and a crucial kinase in maintaining genomic stability. A number of studies have shown the importance of PLK4 in the pathophysiology of cancer. However, cancer-driver mutations have not been identified so far in PLK4. Despite this, its anomalous expression is gaining growing attention in the context of chromosomal instability, tumorigenesis, and poor clinical outcomes across a wide spectrum of cancers.^23^^,^^155^ As research into PLK4 signaling advances, multiple avenues for future studies and therapeutic innovation are emerging. As discussed earlier, PLK4 expression is tightly regulated at multiple levels, for example, transcriptional repression by p53, post-translational degradation via SCF/β-TRCP, and modulation by microRNAs.^33^^,^^35^^,^^36^^,^^39^^,^^43^^,^^155^ Further, evidence suggests that PLK4 may influence EMT, anoikis resistance, and stemness, contributing to metastatic dissemination.^115^^,^^136^ Investigating the epigenetic landscape surrounding PLK4, including DNA methylation, histone modifications, and long non-coding RNAs, as well as PLK4’s interactions with EMT regulators and its role in tumor plasticity, could uncover new therapeutic targets and diagnostic markers. Moreover, understanding how PLK4 modulates cell polarity, motility, and cytoskeletal dynamics may provide insights into its role in invasion and metastasis. The precise molecular mechanisms by which PLK4 regulates primary cilia dynamics in cancer also remain largely unexplored. All these avenues open paths for future investigations.

PLK4 overexpression has been correlated with poor prognosis, chemoresistance, and tumor progression in multiple cancers. The integration of predictive biomarkers such as p53 status, CEP131 phosphorylation, TRIM37 expression, TFEB/TFE3 phosphorylation, miRNA signatures (e.g., miR-338-3p), and POLQ co-expression could enable personalized treatment strategies. Some of the existing PLK4 inhibitors, such as CFI-400945, have demonstrated preclinical efficacy in suppressing tumor growth and inducing apoptosis. Although CFI-400945 was designed to selectively inhibit PLK4 but it has been reported to inhibit other kinases such as PLK1, PLK2 and aurora A/B at higher concentrations.^15^^,^^156^ This is due to structural similarities in the conserved ATP-binding domain across the kinases,^15^ which makes it difficult to design small molecules inhibitors that can interact exclusively with one target and do not give any off-target effects.

Future drug development should focus on structure-guided design and allosteric modulation to enhance specificity and minimize off-target effects. For instance, small-molecule inhibitors that target PLK4’s cryptic polo-box domain, which is a distinct feature of PLK4, may offer novel mechanisms of action. Moreover, dual inhibition of PLK4 and its associated centriolar proteins may offer additional therapeutic advantages in cancer management. For example, in centrinone-induced acentrosomal cells, TRIM37 knockout facilitates cell survival by promoting the formation of acentrosomal spindle assembly with foci enriched with CEP192 and inactive PLK4, which act as MTOCs.^105^ In contrast, TRIM37 overexpression under the same conditions leads to pronounced mitotic defects, primarily due to reduced CEP192 expression.^105^ This context-dependent relationship, driven by changes in TRIM37 and PLK4 levels, highlights a promising therapeutic strategy for tumors exhibiting TRIM37 amplification. In addition, the impact of PLK4 inhibition in drug-resistant cancer models has not been thoroughly investigated, which could provide valuable insights into its therapeutic potential.

Despite promising preclinical data, PLK4 inhibitors remain underexplored in clinical settings. Further studies may incorporate biomarker-guided treatment to identify which patients are most likely to benefit from PLK4-targeted therapies. Additionally, 3D cell cultures, patient-derived organoids, and PDX models can bridge the gap between bench and bedside, enabling more accurate prediction of therapeutic efficacy.

In conclusion, PLK4 stands at the intersection of cell division, genomic stability, and cancer progression. Nonetheless, the mechanisms of PLK4 remain incompletely understood and warrant further research. Continued investigation into the signaling network and therapeutic vulnerabilities of PLK4 holds significant promise for advancing precision oncology and improving patient outcomes. Expanding clinical trials and translational studies will be critical to validate these findings and bring PLK4-targeted therapies closer to clinical application.

## Acknowledgments

This study was supported by the University of Wisconsin Foundation’s Dr. Frederic E. Mohs Skin Cancer Research Chair endowment to N.A. We also acknowledge funding support from the 10.13039/100000002NIH (R01CA261937 to N.A.), and the 10.13039/100000738Department of Veterans Affairs (VA Merit Review Awards I01CX002210 and I01BX005917), and a Senior Research Career Scientist Award IK6BX006041 to N.A.).

## Author contributions

D.M.: writing – original draft, conceptualization, data curation, and visualization. G.C.: conceptualization, and writing – review & editing. K.B.A.A: writing-original draft, and data curation. N.A.: funding acquisition, supervision, and writing – review & Editing.

## Declaration of interests

The authors declare no competing interests.

## Declaration of generative AI and AI-assisted technologies in the writing process

During the preparation of this manuscript the authors used “Co-pilot” to improve the readability and language of the manuscript. We used the prompts such as “rephrase”, “give synonyms for”, etc. for language improvement. After using this tool/service, the authors reviewed and edited the content as needed and take full responsibility for the content of the published article.
